# miR-141 and miR-200c as Markers of Overall Survival in Early Stage Non-Small Cell Lung Cancer Adenocarcinoma

**DOI:** 10.1371/journal.pone.0101899

**Published:** 2014-07-08

**Authors:** Rut Tejero, Alfons Navarro, Marc Campayo, Nuria Viñolas, Ramon M. Marrades, Anna Cordeiro, Marc Ruíz-Martínez, Sandra Santasusagna, Laureano Molins, Josep Ramirez, Mariano Monzó

**Affiliations:** 1 Molecular Oncology and Embryology Laboratory, Human Anatomy Unit, School of Medicine, University of Barcelona, IDIBAPS, Barcelona, Spain; 2 Department of Medical Oncology, Institut Clinic Malalties Hemato-Oncològiques (ICMHO), Hospital Clinic de Barcelona, University of Barcelona, IDIBAPS, Barcelona, Spain; 3 Department of Pneumology, Institut Clínic del Tórax (ICT), Hospital Clinic de Barcelona, University of Barcelona, IDIBAPS, CIBER de Enfermedades Respiratorias (CIBERES), Barcelona, Spain; 4 Department of Pathology, Centro de Diagnóstico Biomédico (CDB), Hospital Clinic de Barcelona, University of Barcelona, IDIBAPS, CIBERES, Barcelona, Spain; 5 Department of Thoracic Surgery, Institut Clínic del Tórax (ICT), Hospital Clinic de Barcelona, University of Barcelona, Barcelona, Spain; H. Lee Moffitt Cancer Center & Research Institute, United States of America

## Abstract

**Background:**

Several treatments in non-small cell lung cancer (NSCLC) are histology-dependent, and the need for histology-related markers is increasing. MicroRNAs (miRNAs) are promising molecular markers in multiple cancers and show differences in expression depending on histological subtype. The miRNA family miR-200 has been associated with the regulation of epithelial-mesenchymal (EMT)/mesenchymal-epithelial transition (MET). EMT involves profound phenotypic changes that include the loss of cell-cell adhesion, the loss of cell polarity, and the acquisition of migratory and invasive properties that facilitates metastasis. A dual role for the miR-200 family in the prognosis of several tumors has been related to tumor cell origin. However, the prognostic role and function of miR-200 family in early-stage NSCLC adenocarcinoma and squamous cell carcinoma (SCC) have not been well established.

**Methods:**

miRNA expression was determined using TaqMan assays in 155 tumors from resected NSCLC patients. Functional studies were conducted in three NSCLC cell lines: H23, A-549 and HCC-44.

**Results:**

High miR-200c expression was associated with shorter overall survival (OS) in the entire cohort (p = 0.024). High miR-200c (p = 0.0004) and miR-141 (p = 0.009) expression correlated with shorter OS in adenocarcinoma – but not in SCC. In the multivariate analysis, a risk score based on miR-141 and miR-200c expression emerged as an independent prognostic factor for OS in the entire cohort (OR, 2.787; p = 0.033) and in adenocarcinoma patients (OR, 10.649; p = 0.002). Functional analyses showed that miR-200c, was related to mesenchymal-epithelial transition (MET) and affected cell migration and E-cadherin levels, while overexpression of miR-141 reduced *KLF6* protein levels and produced an increase of secretion of *VEGFA in vitro* (H23, p = 0.04; A-549, p = 0.03; HCC-44, p = 0.02) and was associated with higher blood microvessel density in patient tumor samples (p<0.001).

**Conclusion:**

High miR-141 and miR-200c expression are associated with shorter OS in NSCLC patients with adenocarcinoma through MET and angiogenesis.

## Introduction

Lung cancer is the most common cause of cancer death, with more than 226,000 new cases in the United States in 2012 [1]. Eighty percent of lung cancers are non-small-cell lung cancer (NSCLC) [2], which has a 5-year survival of only 10% overall and 60–70% in stage I patients, highlighting the need for novel diagnostic and therapeutic strategies. Surgical resection, when possible, remains the only curative treatment for early-stage NSCLC. However, nearly 50% of resected patients experience recurrence and have a dismal prognosis [2]. Several novel treatments in NSCLC are histology-dependent, and squamous cell carcinoma (SCC) responds somewhat differently than adenocarcinoma to certain treatment regimens[3], [4] [5]. However, few histology-dependent prognostic biomarkers are available for routine use in clinical practice, especially in resectable patients.

In recent years, microRNAs (miRNAs) have emerged as promising molecular markers in multiple cancers, including NSCLC [6]. Specific miRNAs have been described as histology-specific prognostic markers for SCC (miR-146b and miR-155) [7] or adenocarcinoma (miR-21) [8].

The miR-200 family is composed of five members located in two different clusters: miR-200a, miR-200b and miR-429 comprise cluster 1(chromosome 1), and miR-200c and miR-141 comprise cluster 2 (chromosome 12). All five miRNAs have been associated with the regulation of epithelial-mesenchymal (EMT)/mesenchymal-epithelial transition (MET) [9]. EMT involves profound phenotypic changes that include the loss of cell-cell adhesion, the loss of cell polarity, and the acquisition of migratory and invasive properties [10]. This process is fundamental for embryonic development and is also involved in tumor invasion and metastasis [11]. The miR-200 family act through their targets ZEB1 and ZEB2 [9] and TGF-β2 [12]. The miRNAs are thus able to enforce the epithelial phenotype through post-transcriptional repression of these genes, allowing the expression of E-cadherin and of polarity factors necessary for the formation of cell-cell junctions. The miR-200 family seems to have a dual role in patient prognosis. Overexpression of the miR-200 family acts as a marker of better outcome in gastric and ovarian cancers [13], [14], [15]. In breast cancer [16] and NSCLC [17] in contrast, high expression of the miR-200 family is associated with shorter survival. In breast cancer, the miR-200 family promotes metastasis through an non-E-cadherin-related mechanism, targeting *SEC23A*, which mediates secretion of metastasis-suppressive proteins[16]. However, the role of high miR-200 levels in NSCLC has not yet been elucidated.

In the present work, we have analyzed the role of members of the miR-200 family in tumors from resected NSCLC patients and correlated our findings with overall survival (OS) after surgery, both in the entire cohort and according to histological subtypes. In addition, we have studied the functional implications of the prognostic markers in NSCLC cell lines.

## Results

### Patients

Table 1 shows the main clinical characteristics for all 155 patients. Median age was 65 years (range, 35–85) and 87% were males. Twenty-one (13.6%) patients had Eastern Cooperative Oncology Group (ECOG) performance status (PS) 0, and 132 (85.2%) had PS 1. Ninety-four (60.6%) patients had stage I disease. Seventy-three (47.1%) patients had adenocarcinoma and 70 (45.1%) SCC. One hundred and thirty-eight (89%) patients were current or former smokers. Twenty (12.9%) patients received adjuvant chemotherapy (16 for stage II or III disease and four for stage I disease with T>4 cm). Median follow-up was 43 months (range, 2-160). After a follow-up of 160 months, 70 (45.2%) patients had relapsed.

**Table 1:** Main patient characteristics.

Characteristic	Value	N = 155, N (%)
Sex	Male	135 (87)
	Female	20 (13)
Age	≤65	74 (47.8)
	>65	81 (52.2)
ECOG Performance Status	0	21 (13.6)
	1	132 (85.2)
	2	2 (1.2)
Disease Stage	I	94 (60.6)
	II	34 (22)
	III	27 (17.4)
Histology	Adenocarcinoma	73 (47.1)
	Squamous cell carcinoma	70 (45.1)
	Others	12 (7.8)
Smoking History	Current smoker	61 (39.3)
	Former smoker	77 (49.7)
	Never smoker	9 (5.8)
	Unknown	8 (5.2)
Type of Surgery	Lobectomy/Bilobectomy	121 (78.1)
	Pneumonectomy	25 (16.1)
	Atypical resection	9 (5.8)
Adjuvant Chemotherapy	Yes	20 (12.9)
	No	135 (87.1)
Recurrence	No	85 (54.8)
	Yes	70 (45.2)
TP53 mutated	Yes	32 (62.6)
	No	97 (20.6)
	Unknown	26 (16.8)

### miR-200 family expression and clinical characteristics

Paired tumor and normal tissue samples were obtained from 155 NSCLC patients. The five members of miR-200 are found in two clusters: miR-200a/b, and miR-429 (cluster 1) and miR-200c and miR-141 (cluster 2). All members of the miR-200 family, except miR-141, were downregulated in tumor compared to normal tissue (miR-200a, p = 0.043; miR-200b, p<0.001; miR-429, p = 0.003; miR-200c, p<0.001) (Figure S1).

Patients with PS 1-2 showed higher levels of miR-429 (p = 0.039) than those with PS 0. Current smokers had lower levels of miR-200a (p = 0.027) and miR-429 (p = 0.032) than never-smokers or former smokers.

### miR-141 and miR-200c as markers of OS

**Figure 1:**
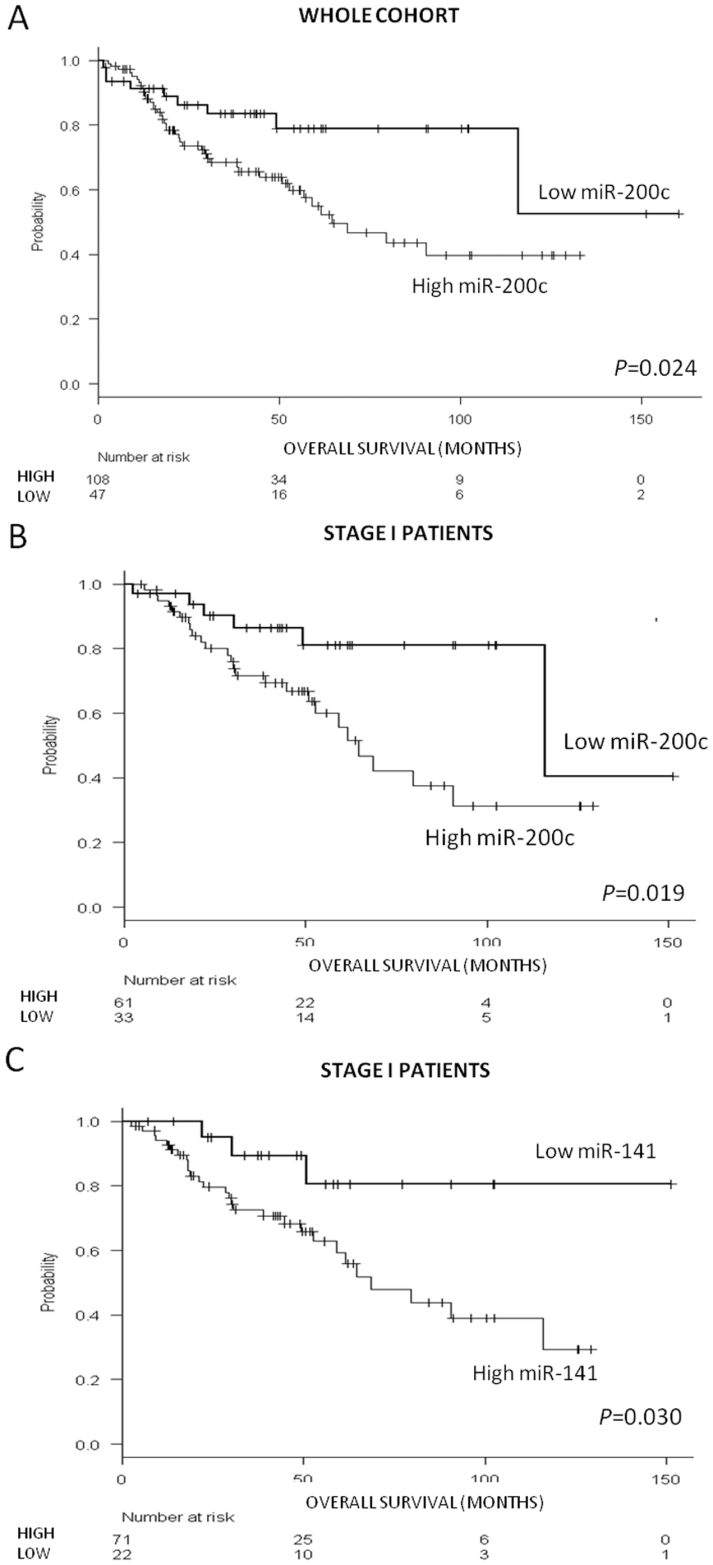
OS analysis in the entire cohort. OS according to **(A)** miR-200c expression levels in the entire cohort (N = 155); **(B)** miR-200c expression levels in stage I patients (N = 94); and **(C)** miR-141 expression levels in stage I patients (N = 94).

Using the cutoffs defined by the Maxstat package of R, we classified patients as having high or low expression levels of the five miRNAs. Among the 155 patients in the entire cohort, those with high levels of miR-200c showed a shorter OS (p = 0.024; Figure 1A). Among the 94 patients with stage I disease, high levels of miR-200c (p = 0.019; Figure 1B) and miR-141 (p = 0.03; Figure 1C) were both associated with shorter OS. No significant differences in OS were identified according to the expression levels of the remaining miRNAs.

### miR-141 and miR-200c in adenocarcinoma

**Figure 2:**
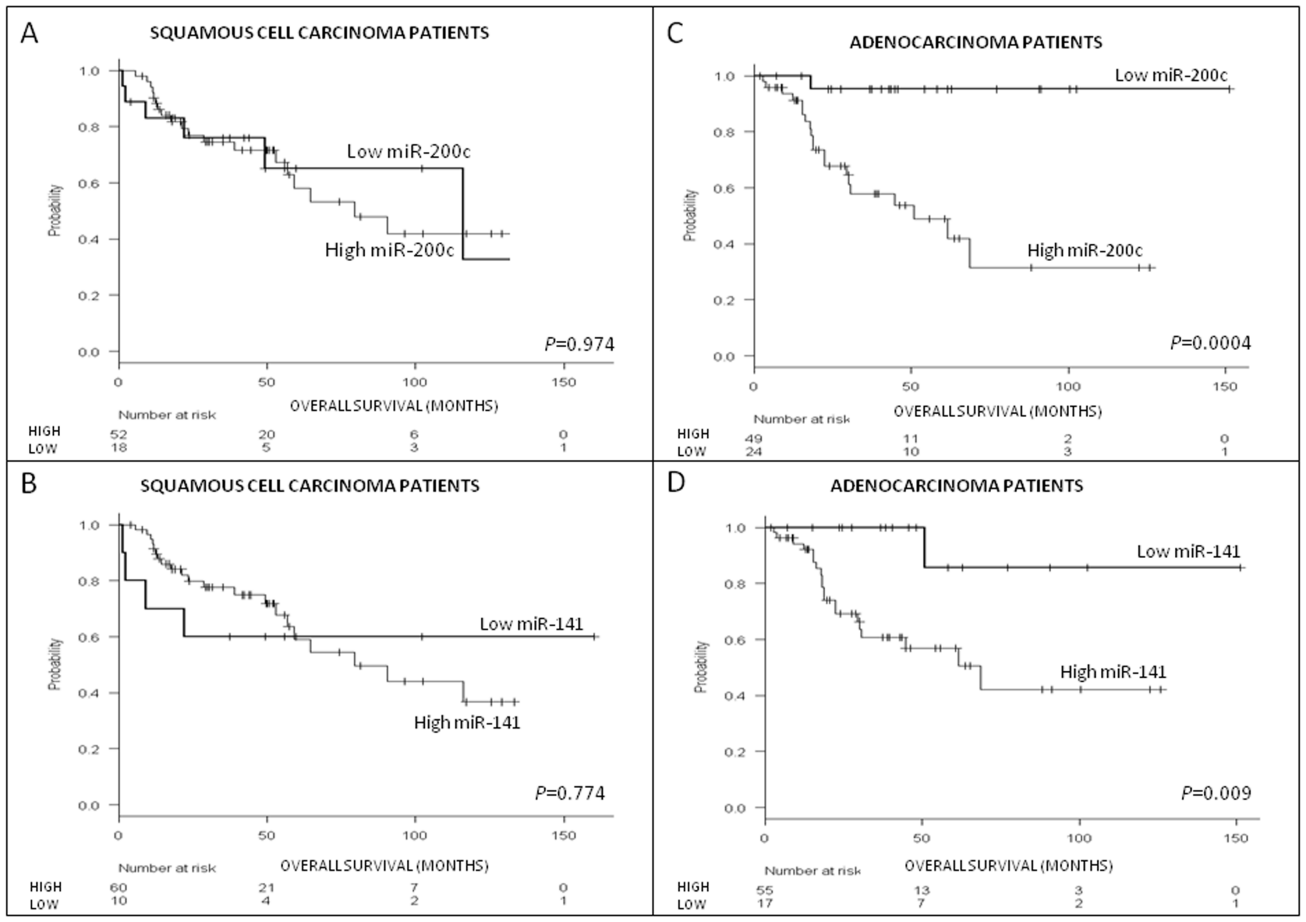
OS analysis by histological subtype. Overall survival according to **(A)** miR-200c expression levels in patients with SCC (N = 70); **(B)** miR-141 expression levels in patients with SCC (N = 70); **(C)** miR-200c expression levels in patients with adenocarcinoma (N = 73); and **(D)** miR-141 expression levels in patients with adenocarcinoma (N = 73).

No differences in OS were observed according to the expression levels of either miR-141 or miR-200c in patients with SCC (Figure 2A and 2B). However, among patients with adenocarcinoma, the miRNAs identified two well-differentiated groups. Mean OS for adenocarcinoma patients with high miR-200c expression was 61.2 months (95% CI = 42.9–79.5), while it was 145.5 months (95% CI = 134.4–156.6) for those with low levels (P<0.001; Figure 2C). Mean OS for adenocarcinoma patients with high miR-141 expression was 71.7 months (95% CI = 44.9–81.6), while it was 136.9 months (95% CI = 110.9-1162.9) for those with low levels (p = 0.009; Figure 2D).

**Figure 3:**
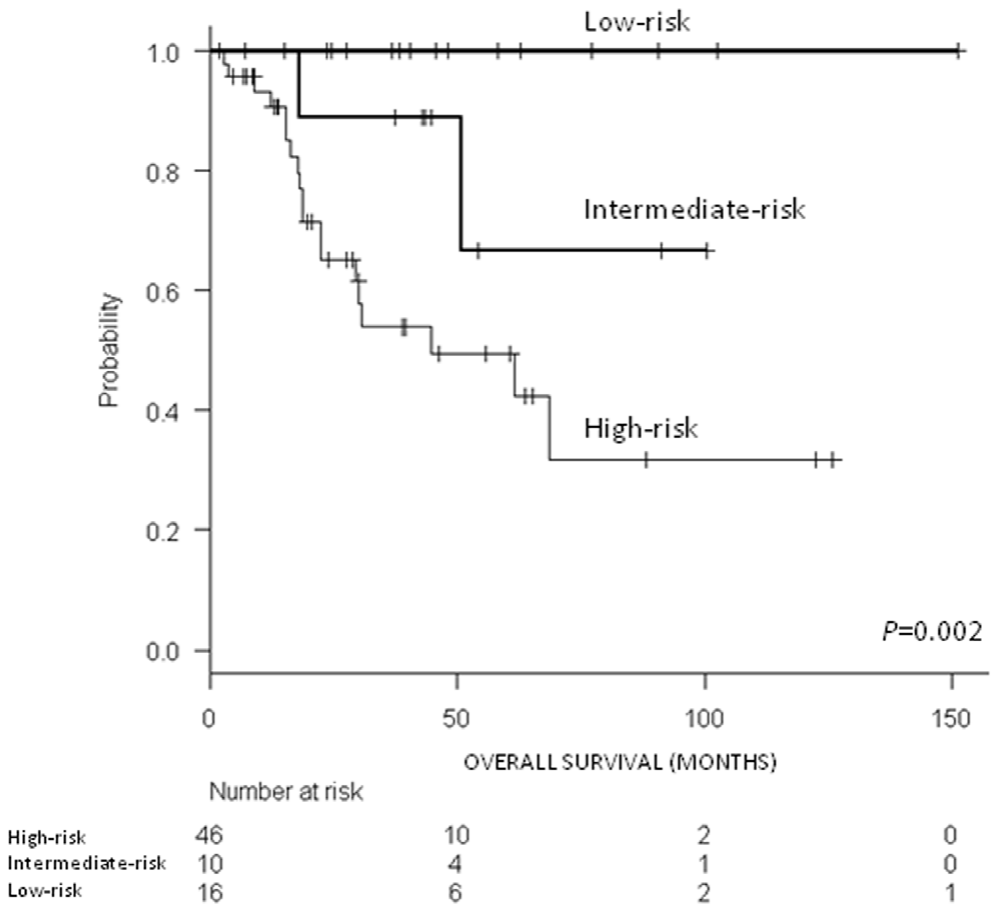
OS in 73 patients with adenocarcinoma according to the miR-141/miR-200c risk score.

We then analyzed the combinatory effect of miR-141 and miR-200c by generating a risk score based on expression levels of both miRNAs. Patients with high levels of both miRNAs were classified as high-risk, those with low levels of both miRNAs as low-risk, and those with other combinations as intermediate-risk. Five year OS was 49.4% for high-risk patients, 66.7% for intermediate-risk patients, and 100% for low-risk patients (p = 0.002; Figure 3).

### miR-200c has a greater impact on cell migration than miR-141

**Figure 4:**
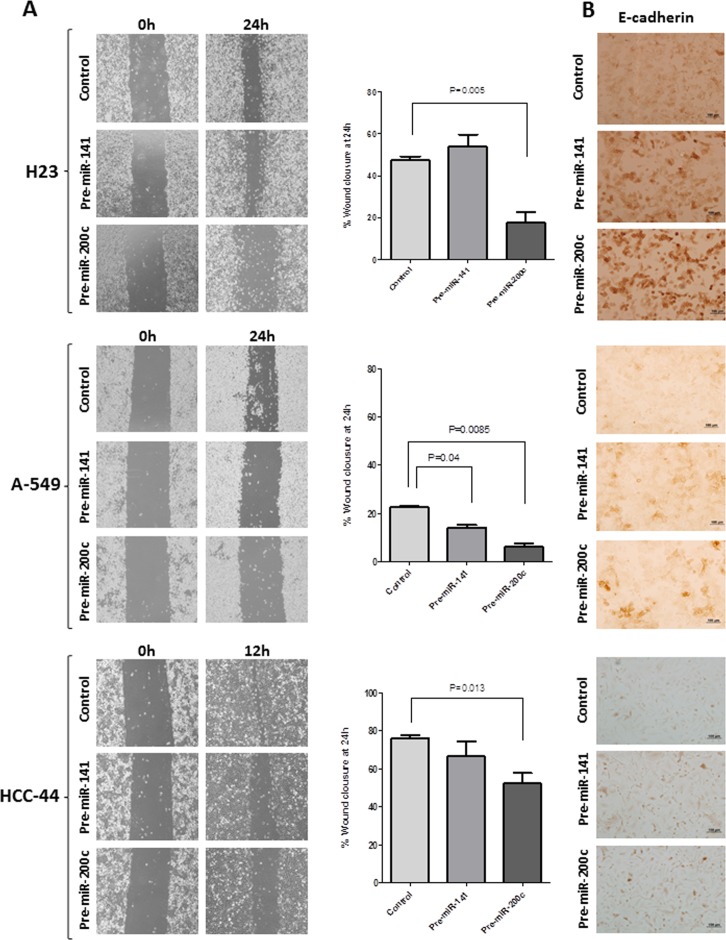
Overexpression of miR-200c affects cell migration. **(A)** After 36 or 48h of transfection with pre-miRNAs in the NSCLC cell lines, cell migration was measured by *in vitro* scratch assay. High levels of miR-200c reduced cell migration in comparison with control in all cell lines (H23, p = 0.005; A-549, p = 0.0085; HCC-44, p = 0.013), while high levels of miR-141 reduced cell migration only in A-549 (p = 0.04). **(B)** E-cadherin levels were evaluated by immunohistochemistry and increased levels were observed in cells transfected with pre-miR-200c in comparison with those transfected with pre-miR-141 or pre-miR-control in all three cell lines.

Cell migration was measured by *in vitro* scratch assay after transfection with pre-miR-200c, pre-miR-141 or pre-miRNA negative control. High levels of miR-200c reduced cell migration in comparison with control in the H23 cell line (p = 0.005), A-549 (p = 0.0085) and HCC-44 (p = 0.013) (Figure 4A). No significant differences were observed for miR-141, except in A-549 (p = 0.043). After transfection, E-cadherin levels were analyzed by immunohistochemistry (Figure 4B) and increased levels were observed in cells transfected with pre-miR-200c.

### miR-141 negatively regulates KLF6, leading to increased VEGFA levels *in vitro*, and is related to higher microvessel density in patient samples

**Figure 5:**
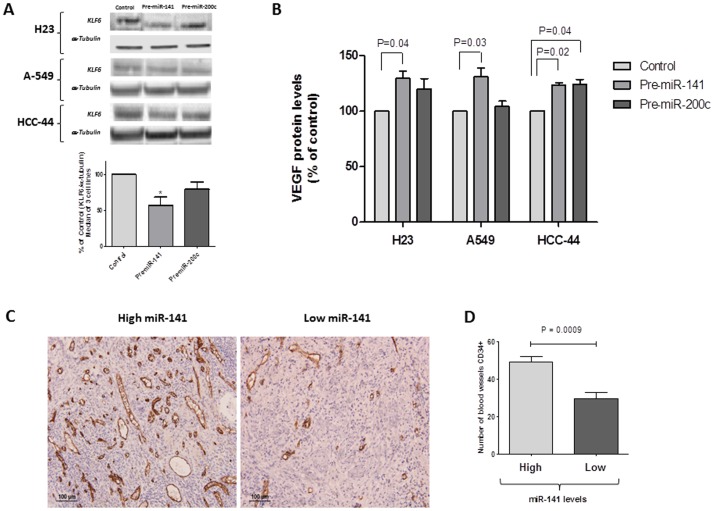
Overexpression of miR-141 negatively regulates KLF6, leading to increased VEGFA levels *in vitro*, and is related to higher microvessel density in patient samples. (A) Immunoblotting of KLF6 in cells transfected with pre-miR-Negative Control or pre-miR-141/200c. miR-141 significantly reduced KLF6 protein level in the three cell lines. (B) After 48 h of transfection with pre-miRNAs in hypoxic conditions, VEGFA concentration in the culture supernatant was measured by ELISA. All results represent the mean ± SEM from 3 independent experiments. (C) A total of 29 adenocarcinoma tumor tissue sections were analyzed by immunohistochemistry with CD34+ as vessel marker; two representative cases with high/low levels of miR-141 are shown. (D) Significant differences in blood microvessel density between adenocarcinomas with high levels of miR-141 and those with low levels of miR-141 were observed (p<0.001).

Since miR-141 overexpression had previously been related to higher blood vessel formation in ovarian tumors of mouse models [18], we examined the potential impact of this mechanism on the prognostic role of miR-141 in NSCLC. Using TargetScan 6.2, we identified KLF6 as a putative target of miR-141 but not of miR-200c. Immunoblotting of KLF6 in H23 cells transfected with pre-miR-141or pre-miR-200c showed that only miR-141 significantly reduced the KLF6 protein levels at 24 h (Figure 5A). Since KLF6 regulates the expression and secretion of VEGFA, a critical angiogenic factor [19], we examined the effect of increasing miR-141 expression levels on *VEGFA* levels. We overexpressed both miR-141 and miR-200c in the H23, A-549 and HCC-44 NSCLC cell lines and treated the cells with DFX to produce hypoxia. After 48 hours, we analyzed the protein levels of VEGFA in the supernatant of these cells. The overexpression of miR-141 produced a mean increase of 28% in release of VEGFA (H23, p = 0.04; A-549, p = 0.03, HCC-44, p = 0.02), while no significant differences were observed for miR-200c, except in the HCC-44 cell line (p = 0.04) (Figure 5B).

We then sought to determine if there exists a relation between miR-141 expression levels and the number of blood vessels in patient samples. After determining the number of blood vessels in tumor samples from 29 patients by immunohistochemistry, using an antibody against CD34+, which is a marker of the blood vessel endothelium (Figure 5C), we classified the tumor samples in two groups: high or low levels of miR-141. The mean number of blood vessels in tumors with high levels was 22% higher than the mean in tumors with low levels (P<0.001; Figure 5D).

### Multivariate analyses

In the multivariate analysis in the entire cohort, we included all the clinical and biological factors with univariate P≤0.1 and the risk score based on miR-141 and miR-200c expression. The miR-141/miR-200c risk score (high-risk vs others) emerged as an independent prognostic marker of shorter OS (OR, 2.787; 95% CI = 1.087-7.148; p = 0.033), together with stage > I and age >65 (Table 2).

**Table 2:** Multivariate analysis for OS in the entire cohort (N = 155) and in the subgroup of patients with adenocarcinoma (N = 73).

ENTIRE COHORT		
OS	OR (95% CI)	*P*
Male sex	2.773 (0.949–8.100)	0.062
Stage >I	2.58 (1.178–5.494)	0.017
Age >65	2.629 (1.374–5.029)	0.003
High-risk miR-141/miR-200c score	2.787 (1.087–7.148)	0.033
		
ADENOCARCINOMA		
OS	OR (95% CI)	*P*
Age>65	3.693 (1.420–9.601)	0.007
High-risk miR-141/miR-200c score	10.649 (2.433–46.608)	0.002

In the multivariate analysis including only adenocarcinoma patients, the miR-141/miR-200c risk score was also an independent prognostic factor for OS (OR, 10.649; 95% CI = 2.433–46.608; p = 0.002) together with age >65 (Table 2).

## Discussion

In the present work, we have found that high miR-141 and miR-200c expression are associated with shorter survival in resected NSCLC adenocarcinoma patients, including those with early-stage disease. Moreover, the combinatory effect of the two miRNAs was an independent prognostic factor for OS. Different mechanisms are involved in the effect of these miRNAs; while miR-141 seems to act through angiogenesis by inhibiting *KLF6* and increasing *VEGFA* levels, miR-200c plays a role in the regulation of MET.

Recently, a phenotypic plasticity has been postulated for transient EMT-MET processes [20]. Induction of MET by overexpression of miR-200 family members is important at a later point in the metastasis process. While EMT allows the cell to migrate from the primary tumor, MET enables it to colonize and produce metastases in distant organs [10], [11]. Thus, both downregulation and overexpression of miR-200 family members have been related to worse prognosis. In order to investigate if the regulation of MET by miR-141 and miR-200c was histology-dependent, we examined their prognostic value in the two major histological subtypes and found both miR-141 and miR-200c were related to OS only in adenocarcinoma patients. It has been observed that NSCLC adenocarcinoma is a more mesenchymal-like tumor type, since it has been observed that vimentin, a marker of mesenchymal cells, was overexpressed in well differentiated adenocarcinomas and in the H23, A-549 and HCC-44 cell lines, but not detected in SCC tissues [21], [22]. In line with our results, miR-200c overexpression as a marker of poor prognosis was previously reported in a cohort of 70 NSCLC patients comprised of both adenocarcinoma and SCC histologies [17], but its prognostic value was not examined in the subgroup of patients with adenocarcinoma. In other tumor models, such as breast cancer, overexpression of miR-200 family members led to increased metastasis [23] and is associated with poor prognosis [16]. In addition, high serum levels of miR-141 have been associated with poor prognosis in colon cancer [24] and high levels of miR-200c with poor prognosis in gastric cancer [25]. In contrast, a work using data from an online TCGA database reported that low levels of miR-200b*, miR-200a and miR-429 were related to shorter OS in a heterogeneous cohort of NSCLC patients that included those with metastatic disease [26].

Since miRNAs modulate the levels of multiple target proteins depending on their sequence, their effect is dependent on the proteins expressed in each cellular type [27]. Both miR-141 and miR-200c are located in the same chromosomal region (12p13.31) and share the same transcription starting site [28], but if we group the miRNAs of the miR-200 family according to the similarity of their seed sequence, we can identify two different clusters – miR-200bc/429 and miR-200a/141 – which are differentiated by a single nucleotide change [29]. Although miR-141 and miR-200c are transcriptionally regulated in the same way, they differ in their targets. When we analyzed the role of miR-141 and miR-200c in EMT/MET in the H23, A-549 and HCC-44 cell lines, only miR-200c influenced EMT in all three cell lines. Overexpression of miR-200c increased the protein levels of E-cadherin and reduced the migration capacity of the tumor cells, as was previously shown by Ceppi *et al* in a different panel of NSCLC cell lines. Ceppi *et al* investigated the expression of miR-200c *in vitro* and *in vivo* and a strong inverse correlation with invasion was detected. Reintroduction of miR-200c into highly invasive/aggressive NSCLC cells induced a loss of the mesenchymal phenotype by restoring E-cadherin and reducing N-cadherin expression, and inhibited *in vitro* cell invasion as well as *in vivo* metastasis formation [30]. Moreover, Pacurari et al. found that miR-200c downregulation in SCC lung tumor samples was correlated with increased levels of DCL1, ATRX and HFE – biomarkers related to EMT [31].

Although miR-141 is a mir-200 family member, it was not involved in EMT/MET. The *in vitro* overexpression of miR-141 was related to reduction of *KLF6* protein levels, producing an increase in the secretion of *VEGFA*. Moreover, tumors with high levels of miR-141 had a higher number of blood vessels. It has been shown that cancer-associated fibroblasts (CAFs) isolated from murine lung adenocarcinomas secreted abundant VEGFA and enhanced tumor cell invasion in coculture studies [32]. When we analyzed the expression of miR-141 in tumor and paired normal tissue, we did not observe significant differences in expression levels, leading us to speculate that the prognostic role of miR-141 may be related to its expression in CAF cells rather than in tumor cells. The overexpression of miR-141 would lead to overproduction of VEGFA and increased neoangiogenesis, which have previously been related to prognosis in NSCLC [33].

## Materials and Methods

### Study population and ethics statement

One hundred and fifty-five adult patients diagnosed with NSCLC who underwent complete surgical resection at Hospital Clinic in Barcelona, Spain between March 1996 and December 2009 were included in the study. Approval for the study was obtained from the center's institutional review board, and written informed consent was obtained from each participant in accordance with the Declaration of Helsinki.

### RNA extraction and miRNA analysis

Total RNA and miRNA detection was performed from FFPE tumor tissues as previously described [34]. MiRNA detection was performed using commercial assays (TaqMan MicroRNA assays, Appplied Biosystems).

### Western Blot analysis

Western Blot analysis was performed as previously described [35] using the following primary antibodies: KLF6 sc-7158 (Santa Cruz Biotechonology) and α-tubulin (Sigma).

### Immunohistochemistry

The immunohistochemical assay, were performed as previously described [36] using a Flex Monoclonal Mouse Anti-Human CD34 Class II clone QBend 10 Ready-to-Use (Dako) and Monoclonal Mouse Anti-Human E-Cadherin Clone NCH-38 (Dako).

### Blood vessel quantification

Four independent areas were selected under a 40X field, and a 200X field (0.785 mm^2^ per field) and used to count CD34-positive vessels in each of these areas. Two independent pathologists examined the slides, and the average of four 200X field counts of CD34-positive vessels was recorded.

### Cell lines, miRNA transfection and VEGF quantification

H23 (American Type Culture Collection) and HCC-44 (DSMZ) cells were cultured in RPMI 1640 (Invitrogen) containing 10% fetal calf serum (Invitrogen). A-549(DSMZ) cells were cultured in DMEM (Invitrogen) containing 10% fetal calf serum.

One day before transfection, 7×10^4^ cells were seeded in 6-well plates. The following day, cells were transfected with 100nM pre-miR-141/200c or pre-miR-Negative Control#2 using Lipofectamine 2000 (Invitrogen). At 24 h post-transfection, cells were treated with 400 µM desferrioxamine to induce hypoxic conditions. After 24 h incubation, VEGF concentration in supernatants was measured in triplicate using the VEGF Human Elisa Kit (ab100662, Abcam).

### Cell migration analysis

Cell migration was measured by *in vitro* scratch assay [37]. 5*10^5^ cells were plated in a 12-well plate one day before transfection with pre-miRNAs. Twenty-four hours after transfection, the cell monolayer was scraped in a straight line to create a “scratch” with a p100 pipet tip. The migration distance (µm) was assessed at 36 h (HCC-44) or at 48 h (H23 and A-549) after transfection using cellSense Entry 1.7 software (Olympus).

### Statistical analyses

OS was calculated from the time of surgery to the date of death or last follow-up. Kaplan-Meier curves for OS, with their 95% confidence intervals (CIs), were drawn and compared by means of a log-rank test. All factors with a p-value<0.1 in the univariate analysis were included in the Cox multivariate regression analyses for OS.

Paired t-test was used to compare expression levels of miRNAs between tumor tissue and paired normal tissue. Non-paired t-test was used to compare differences between two groups. Optimal cutoffs of miRNA expression data for OS were assessed by means of maximally selected log-rank statistics using the Maxstat package (R package). The applicability of these cutoffs was confirmed by the Kaplan-Meier test. All statistical analyses were performed using PASW Statistics v18 (SPSS) and R v2.8.1. The level of significance was set at ≤0.05.

## Supporting Information

Figure S1 **Expression levels of miR-200 family members obtained from 155 NSCLC tumor and paired normal tissue.** **(A)** Cluster 1: miR-200a, miR-200b, and miR-429. **(B)** Cluster 2: miR-141 and miR-200c.
(TIF)
