# Supplementary figures and images for: miR-141 and miR-200c as Markers of Overall Survival in Early Stage Non-Small Cell Lung Cancer Adenocarcinoma
Source: PLoS One. 2014 Jul 8;9(7):e101899. doi: 10.1371/journal.pone.0101899 (PMC4087018; doi:10.1371/journal.pone.0101899)

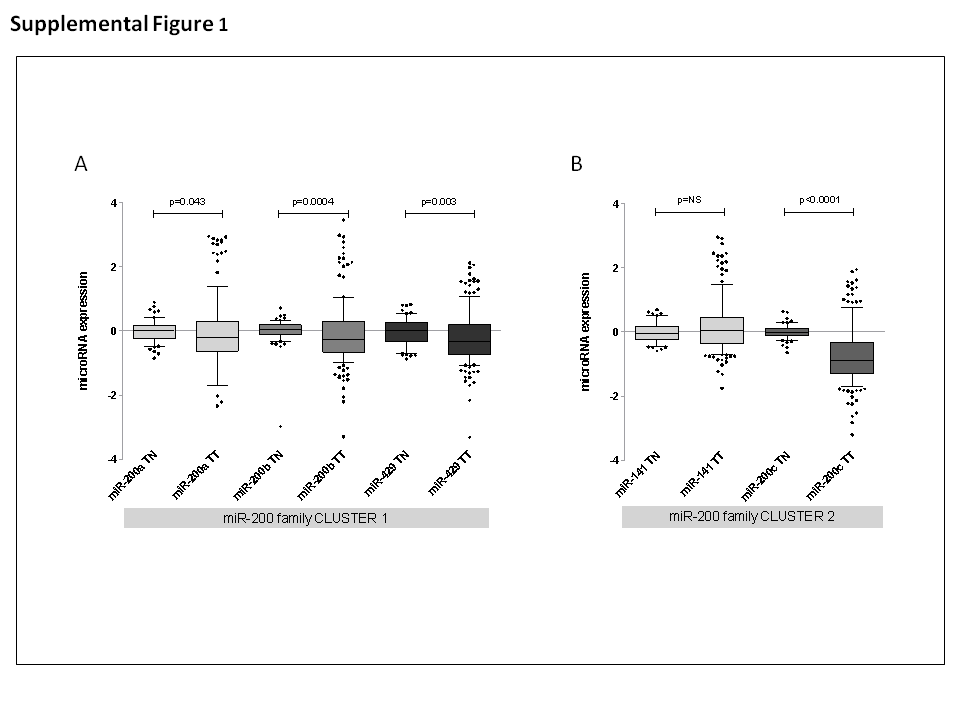

Supplement: Figure S1 — Expression levels of miR-200 family members obtained from 155 NSCLC tumor and paired normal tissue. (A) Cluster 1: miR-200a, miR-200b, and miR-429. (B) Cluster 2: miR-141 and miR-200c. (TIF) [file pone.0101899.s001.tif]
